# Sweetened beverage taxes: Economic benefits and costs according to household income

**DOI:** 10.1016/j.foodpol.2022.102277

**Published:** 2022-06-02

**Authors:** Jessica C. Jones-Smith, Melissa A. Knox, Norma B. Coe, Lina P. Walkinshaw, John Schoof, Deven Hamilton, Philip M. Hurvitz, James Krieger

**Affiliations:** a University of Washington, School of Public Health, Department of Health Systems and Population Health, 3980 15^th^ Ave NE, Seattle, WA 98195, United States; b University of Washington, School of Public Health, Department of Epidemiology, 3980 15^th^ Ave NE, Seattle, WA 98195, United States; c University of Washington, Department of Economics, 305 Savery Hall, Seattle, WA 98195, United States; d University of Pennsylvania, Perelman School of Medicine, Department of Medical Ethics and Health Policy, 423 Guardian Drive, Blockley Hall, Philadelphia, PA 19104, United States; e University of Washington, Center for Studies in Demography and Ecology, 206 Raitt Hall, Seattle, WA 98195, United States; f University of Washington, Urban Form Lab, 45th Street, Suite 535, Seattle, WA 98105, United States; g Healthy Food America, PO Box 22260, Seattle, WA 98122, United States

**Keywords:** Sugar sweetened beverages, Beverage tax, Sin tax, Regressive tax, Obesity

## Abstract

Taxing sweetened beverages has emerged as an important and effective policy for addressing their overconsumption. However, taxes may place a greater economic burden on people with lower incomes. We assess the degree to which sweetened beverage taxes in three large US cities placed an inequitable burden on populations with lower incomes by assessing spending on beverage taxes by income after taxes have been implemented, as well as any net transfer of funds towards lower income populations once allocation of tax revenue is considered. We find that while lower income populations pay a higher percentage of their income in beverage taxes, there is no difference in absolute spending on beverage taxes per capita, and that there is a sizable net transfer of funds towards programs targeting lower income populations. Thus, when considering both population-level taxes paid and sufficiently targeted allocations of tax revenues, a sweetened beverage tax may have characteristics of an equitable public policy.

## 1. Introduction

Excessive consumption of sweetened beverages is an important contributor to poor diet quality, weight gain, diabetes, cardiovascular disease, and poor oral health (Malik et al., 2019; Luger et al., 2017; Chi and Scott, 2019; Malik et al., 2010). Taxing sweetened beverages has emerged as an important policy for addressing overconsumption of sweetened beverages, with multiple studies showing that taxes substantially reduce purchasing of taxed beverages (Roberto et al., 2019; Silver et al., 2017; Powell and Leider, 2020; Cawley et al., 2019), while a few studies show no impacts on purchasing (Bollinger and Sexton, 2018). Estimated impacts on consumption have been mixed (Cawley et al., 2019; Cawley et al., 2020; Zhong et al., 2018; Lee et al., 2019). A recent *meta*-analysis of 10 studies of taxes in the US found that demand for sugar sweetened beverages declined by 20% with a calculated price elasticity of –1.5. After correction for cross-border shopping, the elasticity was –1.1 (Powell et al., 2021). In the US, seven local jurisdictions and the Navajo Nation have implemented sweetened beverage taxes, as have at least than 45 nations (Popkin and Ng, 2021).

However, a variety of stakeholders have questioned whether sweetened beverage taxes are equitable, given concerns that consumption taxes may place a greater economic burden on people with lower incomes. Specifically, at the same volume of consumption, for a volume-based tax, lower-income households pay a greater proportion of their income on sweetened beverage excise taxes as compared to higher-income households. Furthermore, consumption in the absence of a tax tends to be higher among populations with lower incomes.

There are three factors that may affect economic equity aspects of sweetened beverage taxes. First, if these taxes result in a larger decrease in sweetened beverage purchases among lower-income compared to high-income populations, the absolute amount of tax paid post-tax may not differ by income. Second, the absolute size of the differential tax paid by income may be small. Most modeling studies of US tax scenarios reported small predicted differences in absolute amounts paid in beverage taxes, with the largest being approximately US$5 per capita per year (Backholer et al., 2016). Third, any amount of tax paid disproportionately by lower-income groups should be placed in the broader context of overall tax policy economic impacts, which include tax revenue allocation. Multiple US cities with taxes earmarked the tax revenues to invest in lower-income communities (Falbe, 2020).

Thus, the tax policy may promote equity if the tax policy as a whole produces a net transfer of resources from higher-to lower-income populations.

While several studies have modeled the predicted difference in spending on sweetened beverage taxes by income, no studies to our knowledge have quantified the real-world estimated amount of tax paid according to household income nor compared estimated population-level tax paid by income to benefits received from tax revenues. Our objectives were to quantitatively assess the equity aspects of sweetened beverage taxes by (1) estimating the difference in the amount of tax paid per capita according to household income and (2) examining the aggregate net economic burden of these policies, considering the combined effects of tax payments and investments of tax revenues.

## 2. Empirical application

### 2.1. Overview

Our primary outcomes were (1) per capita annual spending on the beverage tax, stratified by household income, in absolute terms and as a proportion of income, (2) total annual allocation of tax revenues to programs serving lower-income populations, and (3) the net transfer of funds by income group.

Briefly, we used data on annual household beverage purchases in three US cities with taxes - Philadelphia, Seattle, and San Francisco - to estimate annual per capita spending on the tax and the proportion of household income spent on the tax, stratified by income group. We estimated aggregate amounts paid in beverage taxes by each income group by weighting the data to be representative of each city and using city income composition to find total tax paid in aggregate by each income group. In a separate process, we collected information on annual allocations of beverage tax revenues in each of the three municipalities to calculate the aggregated amount of allocations targeting lower-income populations. We compared this to the aggregated amount of taxes paid to describe the net economic transfer of funds between lower-income and higher-income populations.

### 2.2. Sweetened beverage tax policies

We included Philadelphia, San Francisco, and Seattle in our analysis because annual consumer beverage purchase data were available for these three cities from among the seven US cities that have implemented taxes. Each city imposed volume-based excise taxes on beverage distributors. Philadelphia implemented its 1.5 cents per ounce tax on beverages sweetened with sugar or artificial sweeteners in 2017. San Francisco’s 1 cent per ounce on sugar-sweetened beverages tax took effect in 2018. Seattle implemented a 1.75 cent per ounce tax on sugar-sweetened beverages in 2018.

### 2.3. Study population & data sources

The study population consisted of households in the three cities that recorded household purchases for either Nielsen’s Homescan Consumer Panel or Numerator’s OmniPanel. Our final sample included a total 1,141 households, with 585, 212, and 344 households in Philadelphia, Seattle, and San Francisco, respectively. Our primary analysis combined the data from these two panels to maximize sample size. These data are key for our purpose of estimating total annual estimated tax paid since they aim to record all beverages purchased at food stores in households over the course of a year.

#### 2.3.1. Nielsen Homescan consumer panel

Nielsen enrolls a longitudinal sample of households that record all packaged food and beverages they purchase using mobile scanners. The Nielsen dataset includes each product purchased, purchase date, UPC code, purchase location, price, coupon use, and quantity.

#### 2.3.2. Numerator OmniPanel

The Numerator OmniPanel also enrolls a longitudinal sample who scan or send pictures of all their food and beverage receipts. Receipts typically include date of purchase, GTIN (a product code that can be converted to a UPC), product description, quantity purchased, and price paid. The OmniPanel data were missing beverage volume for 48% of taxed beverage purchases. We used a hot-deck imputation process to impute missing volumes. In a test of a random 15% of the observations for which we had non-missing data, the median difference between imputed and actual volume was zero ounces. The imputation process and results are detailed in the Supplemental Materials.

Both data sources collect self-reported information on household income (panelists chose from a set of income ranges; see Supplemental Materials for ranges), race/ethnicity of head of household, household composition, and other demographic data. We converted income values into income as a percent of the US federal poverty level (%FPL) (U.S. Department of Health and Human Services, 2021) by assigning the midpoint value of the income category bounds and dividing by the FPL according to household size. We further aggregated these into three categories: lower (≤200% FPL), middle (>200–400% FPL) and higher income (>400% FPL)).

A limitation of both panels is that they do not collect food or beverages purchased from restaurants.

We analyzed data from the first full year after the tax was implemented in each city. We determined the tax status of each beverage purchased in each city using UPC, GTIN or manual searching based on the item’s name and description and comparing this to each city’s tax regulations.

#### 2.3.3. Tax revenue allocations

We collected total tax revenues for the most recent fiscal year available (Seattle calendar year 2018, San Francisco fiscal year 2020 (July 2019-June 2020) and Philadelphia fiscal year 2021 (July 2020-June 2021)). Data were obtained from publicly available documents including contracts, city budgets, reports, and websites. To supplement publicly available documents, we contacted representatives in each city to obtain additional information. Data included: program name, description of program objectives, funded activities and organization receiving funds, dollars allocated, and target population and geographic area (and user demographics if available).

#### 2.3.4. American Community Survey

We used American Community Survey (ACS) 5-year estimates for 2017 and 2018 to weight our sample to be representative at the household-level for each city. We additionally used data from ACS 2017 to determine the proportion of lower-income households (defined as percent with incomes below 200% FPL) in the geographic areas within cities receiving revenue allocations.

### 2.4. Measures and statistical analyses

#### 2.4.1. Estimated tax paid

##### Per capita annual spending on the beverage tax.

We summed the total ounces of taxed beverages purchased over the entire year for each household and multiplied this by the city-specific amount of the tax per ounce to obtain the total amount spent. We then calculated per capita spending by dividing this amount by the number of people in the household. We assumed that distributors passed through 100% of the tax to consumers, which is common practice in similar work and further justified based on pass-through rates close to 100% in each of these three cities (Cawley et al., 2019; Falbe et al., 2020; Jones-Smith et al., 2020; Urban Institute & Brookings Institution, 2021).

##### Proportion of household income paid on the beverage tax.

To obtain this quantity, we divided the total amount spent on the tax per household by the midpoint of household income category.

##### Statistical analyses for tax paid by income.

All analyses use raking weights to weight the populations to be representative of key characteristics of the cities from which they are drawn based on household head age, race, income, education; household size; and presence of children in the household.

For statistical testing of differences in these outcomes by income, we used two models for each of our primary outcomes. The first set of models used ordinary least squares regression models of log-transformed per capita spending on the tax regressed on income category to test for differences in spending by income. Log-transformation of the spending outcomes was used due to the skewed distribution and regression diagnostics (Manning and Mullahy, 2001). The second set of models used the original form of the outcome variables rather than log-transformed to produce mean spending by income. Robust standard errors were used in all models to account for heteroskedasticity. A p-value of < 0.05 was considered statistically significant.

In secondary descriptive analyses, we examined total spending on taxed beverages, rather than just the tax itself. Spending on taxed beverages was derived from a separate variable—price paid for each beverage purchase. We assessed whether the relationships between income group and spending on beverages themselves were consistent with those seen for income group and spending on the tax.

#### 2.4.2. Net transfer

##### Aggregate beverage tax paid by income.

For each city, we used the mean spending on the tax multiplied by the population in each income group to estimate the ratio of per capita spending on the tax by the lower-income population to spending by the higher-income population. For this analysis, we combined the middle and higher-income categories together (≥200% FPL) to use a dichotomous definition of income groups to match the way that cities target program spending (described further below). We used means rather than medians or back-transformed regression coefficients in order to better capture the full range of spending within each income group. The ratio was then used to estimate the total spending by each income group in the city based on the total reported revenues collected.

##### Amount of tax revenue collected that is targeted to programs serving lower-income populations.

We collected data to describe the proportion of people served by each sweetened beverage tax-funded program who lived in households with incomes less than 200% FPL. When available, we obtained program-level demographic data for participants actually served by the program. If not available, we used program eligibility requirements. If those were not available, we used a statement of the target population for the program. If none were available, we attempted to obtain demographic data for the site hosting the tax-funded program. If none of these sources were available, we used ecologic income data for the area served by the program. For programs that specified a targeted neighborhood or had a city-wide focus, we used 2017 ACS ZCTA- or city-level household income data to describe the proportion of residents living in households with incomes less than 200% FPL. For sites that did not specify a target area, we aggregated income data from the 2017 ACS for all block groups within a 0.5-mile buffer area around the program site. To determine the amount of revenues targeted to people with lower incomes, we multiplied the revenue allocated to each program by the proportion of participants from lower-income households and then summed these revenues to obtain the total revenues benefitting people with lower incomes. We did not include revenues allocated for administration or evaluation in the calculation of revenues targeted towards lower-income populations.

##### Net transfer.

To calculate the net transfer of funds to lower-income populations, we subtracted the aggregate estimated amount spent on the tax by the lower-income population from the amount of tax revenues spent on programs targeting lower-income populations. If this number is positive, it indicates an aggregate transfer from higher-income groups to lower-income groups, and vice versa if it is negative.

##### Sensitivity Analyses.

We present analyses for Nielsen and OmniPanel separately in Supplemental Materials.

## 3. Results

### 3.1. Sample characteristics

Table 1 displays the weighted sample characteristics by city. Unweighted sample characteristics can be found in Supplemental Table 1. Estimated mean income was lowest in Philadelphia and highest in San Francisco. Income categorized according to %FPL followed a similar pattern, with Philadelphia having a larger share of the population < 200% of the poverty line as compared to Seattle or San Francisco (36% vs 19% and 17%, respectively). Racial composition also differed across the cities, with the proportion of the population identified as White being highest in Seattle (63%) compared to San Francisco (40%) and Philadelphia (35%). Philadelphia had a larger proportion of the population identifying as Black (41%) as compared to Seattle (6.8%) or San Francisco (5.5%). Per capita volume of taxed beverage purchases was higher in Philadelphia (mean (SE): 1,957oz (150)) (where artificially sweetened beverages were also taxed) compared to Seattle (mean (SE): 772oz (128)) and San Francisco (mean (SE): 799oz (113)). In Philadelphia and Seattle, there was a graded relationship whereby per capita volume purchased was highest in the lowest-income population and lowest in the highest-income populations (Philadelphia: lower-income: 2069oz (299), middle: 1986oz (225), higher: 1803oz (237); Seattle: lower-income: 1085oz (410), middle: 751oz (220), higher: 681oz (147)). On the other hand, in San Francisco, the gradient was in the opposite direction—the highest-income population had the highest mean volume purchased and the lowest-income population had the lowest (San Francisco: lower-income: 546oz (125), middle: 650oz (137), higher: 902oz (161)). However, the standard errors in all three places suggest differences in means across income groups would not be statistically significant. Per capita spending on beverages followed a similar pattern to volume in each city.

### 3.2. Annual per capita spending on the beverage tax according to income

Table 2 displays the coefficients from the regression models of spending and logged spending on the beverage tax by income. For both the original and logged specifications of per capita spending, the estimates of differences by income are relatively small and, within cities, income differences are not statistically significantly different from each other. For example, the differences by income in the non-logged models are approximately $4 per capita per year in Philadelphia between higher- and lower-income groups, $7 per capita per year for Seattle between higher and lower, and $4 between higher- and lower-income in San Francisco, with the higher-income group paying more in this case. Estimates from the logged models suggest even smaller differences.

### 3.3. Proportion of household income spent on beverage taxes annually, according to income

Spending on beverage taxes as a proportion of income was significantly higher for the lowest-income group as compared to the middle- and the highest-income group in all three cities in both the non-logged and logged outcome models (Table 3).

Additionally, the middle-income group spent a statistically higher proportion of their income on the beverage tax as compared to the highest-income group in all three cities. The proportions of income spent on taxes and taxed beverages were smaller in Seattle and San Francisco, consistent with the pattern of amounts paid for beverages and taxes. The relative difference by income was large (deriving from the differences in the denominator (income)); however, the magnitude of the proportions of income spent on taxes was generally small in populations with lower-income in all three cities (Philadelphia: 0.50% (95% CI: 0.32, 0.68); Seattle: 0.20% (95% CI: 0.045, 0.35); San Francisco: 0.06% (95% CI: 0.039, 0.086)) (back-transformed values from models with logged outcomes are each lower than these values).

Our supplemental analyses examining the outcomes separately by data source (Nielsen or OmniPanel) were similar in terms of direction and statistical significance with the exception that some of the associations were not statistically significantly different when using Nielsen alone (Supplemental Tables 2 & 3).

### 3.4. Tax revenues and net transfers

Table 4 displays the estimated mean per capita contribution to the beverage tax revenue for the lowest-income group (<200% FPL) and the higher-income group (≥200% FPL). In all cities, the proportion of the population with incomes < 200% FPL is smaller than the proportion with incomes ≥ 200 %FPL, resulting in the higher-income group paying a larger share of the aggregate tax paid in each city, with the differences being more marked in Seattle (72%) and San Francisco (85%) compared to Philadelphia (52%). Tax revenues were also targeted to lower-income households, which is especially notable in Philadelphia (70%) compared to Seattle (56%) and San Francisco (55%). In all cities, there was a positive net transfer of funds from the higher-income population to the lower-income population. Specifically, we found a net transfer towards the lower-income population of $16.4 million in Philadelphia, $6.3 million in Seattle, and $5.3 million in San Francisco. This net transfer represents 22% of the revenues collected in Philadelphia, 28% of the revenues collected in Seattle, and 40% of the revenues collected in San Francisco.

## 4. Policy implications

### 4.1. Findings in context and policy implications

We assess the equity attributes of sweetened beverage taxes by comparing the relative estimated amounts of tax paid and net aggregate transfer of tax revenues - considering taxes paid and benefits received from programs funded by tax allocations - across income groups in three US cities. While we found that the proportion of household income spent on beverage taxes was highest among lowest-income populations, we did not find any statistically significant differences in annual absolute dollar amounts spent on beverage taxes per capita by income level. We additionally found a sizable net transfer of tax revenues towards lower-income populations when comparing estimated population-level amount of taxes paid to aggregate amount of tax revenues allocated towards lower-income communities. This is the first study to our knowledge to use real-world data about sweetened beverage taxes to estimate tax economic equity impacts. Prior studies have modeled simulations of hypothetical taxes.

Our finding that the average annual per capita amount of beverage tax paid was largely similar across income groups is consistent with estimates from some, but not all, modeling studies. Most modeling studies of US tax scenarios reported small estimated predicted differences in absolute spending on beverage taxes. Lin and colleagues simulated a 20% excise tax and categorized income as above or below 185% of FPL. They found negligible differences of approximately one dollar per year in per capita amount paid towards the tax by income group (Smith et al., 2010). Zhen and colleagues simulated a tax of 0.5 cent per ounce effective tax rate and came to similar conclusions (Zhen et al., 2014).

Our estimates for the amount of beverage tax paid are reasonable when compared to two studies using the nationwide Nielsen data (i.e., not limited to jurisdictions with the tax) that estimate that a 0.5 cent tax on sugar-sweetened beverages would result in an annual per capita tax payment of approximately $6.50 for those with incomes < 185% FPL. Our estimates for San Francisco are closest to this number. San Francisco has the lowest tax rate (1 cent per ounce) of our three cities, but also likely has lower average consumption of sweetened beverages compared to the national average based its population composition. Our estimates for Philadelphia are higher than this simulation, which also seems reasonable given the higher tax rate in Philadelphia and the fact that Philadelphia’s tax includes artificially sweetened beverages as well. Our estimates on tax paid is approximately twice as high for Seattle; this also seems reasonable due to a much higher tax of 1.75 cents per ounce paired with a lower level of consumption. While our numbers underestimate total beverage spending because they do not capture restaurant spending, the missing purchases are likely higher among higher-income populations who spend more on food away from home (McDowell et al., 1997; Saksena et al., 2018). Additionally, in order to change the conclusions on the net transfer, the differential omission would need to be large (for example, in Philadelphia, it would require an underestimate of an additional $23 per capita in beverage tax spending among the lower-income and no underestimate among the higher-income group).

Our study found that the lowest-income populations spend a larger proportion of their household income on beverage taxes compared to middle- and higher-income populations. For the lowest-income group, our estimates range from 0.06% of income in San Francisco to 0.50% of income in Philadelphia. These are likely underestimates of the exact proportions of income paid since our data sources are likely not capturing all expenditures on the tax as evidenced by the analysis of total aggregate revenues collected. Previous simulation studies using US-wide data mentioned above estimate a range of 0.1% to 1% of income paid to beverage taxes, and similar to our findings.

In all three cities, once we consider the allocation of the tax revenues, in aggregate, a sweetened beverage taxes appears to have characteristics of an equitable public policy. The dollar amount of tax-revenue-funded programs targeted towards people with lower-incomes is greater than the amount lower-income populations pay in taxes. There is a redistributive effect, with a net transfer of taxes collected from middle- and higher-income populations to programs targeted to lower-income populations. This net effect is the result of lower-income populations paying a similar or lower per capita amount in beverage taxes compared to higher-income populations while comprising a smaller proportion of the population, combined with targeted tax allocation. Despite the differences in population demographics, level of sweetened beverage consumption, beverages taxed, tax rates, and uses of the revenue, our findings on the economic equity aspects of tax policy are largely consistent across the three cities.

The progressive-leaning net transfer of sweetened beverage taxes depends on the ability to allocate revenues to programs that benefit people with lower incomes. This is most readily accomplished by dedicating tax revenue by law (also called earmarking) to such programs rather than placing them in a jurisdiction’s general fund. All US states have the authority to dedicate tax revenues, but this authority varies across local US jurisdictions. Similarly, for countries globally, if a taxing jurisdiction cannot earmark sweetened beverage tax revenues to target them to people with lower incomes, the degree to which there will be a progressive net transfer at the aggregate level will be determined by the extent to which general revenues in that jurisdiction are targeted to benefit lower-income populations. The generalizability of our findings is therefore limited to places in which it is acceptable and legal to earmark tax funds for specific purposes.

Taxing artificially sweetened beverages has been proposed as a measure to decrease the regressive burden of beverage taxes since higher-income populations tend to consume more of these beverages compared to lower-income populations (Kane and Malik, 2019). Contrary to this expectation, Philadelphia taxes artificially sweetened beverages and yet its highest-income group did not pay significantly more in annual per capita beverage tax than its lowest-income population. This is consistent with recent “optimal tax” literature (Allcott et al., 2019), which suggests that taxing artificially sweetened beverages will not lead to less regressivity unless the internalities (particularly the negative health effects) of artificially sweetened beverages are as bad as the internalities of sugar-sweetened beverages.

## 5. Limitations

This study has several limitations. To increase sample size, we combined two data sources that use different sampling strategies and different measurement techniques to capture annual spending on taxed beverages. Additionally, neither data source captures beverages purchased at restaurants or other food away from home (such as coffee shops or gas stations), so our estimates of total spending will be underestimates. Our best estimates suggest that we captured 45–66% of the total tax paid. However, we believe the differential by income will be conservative since lower-income groups tend to spend less on food away from home compared to higher-income groups.[28] We were likely underpowered to detect small-sized effects in the absolute per capita spending on the tax. However, the biggest difference in estimated spending were $4–7 per capita per year (with most differences smaller than this) which is arguably a small annual difference in spending by income, regardless of whether it is or is not statistically significantly different.

Beverage volume was missing for a sizable portion of the OmniPanel observations, requiring imputation. The imputation technique performed well with the median difference between true volume and imputed volume being zero ounces. There are two additional limitations to the data that should be acknowledged: income was collected in categories rather than a continuous measure. This could result in misclassification in %FPL categories; the data did not identify each household members’ purchases separately, so instead, we had to assume equal spending across household members in our calculation of per capita spending. We used a pass-through rate of 100% of the tax for our estimates of spending on the tax, although estimates of pass-through in each of these places has varied. The pass-through rate in our study will impact the total estimated amount paid, however because the rate is applied as a constant to volume and the same rate is assumed for lower and higher income populations, a difference in pass-through would not impact the estimated relative difference in amount paid by lower versus higher income. That said, assuming 100% pass-through provides an upper bound on the estimates for the aggregate amount of tax paid by consumers. A lower pass-through rate means beverage companies contribute a portion of tax revenues collected and the aggregate amount contributed by consumers would be lower. Because total revenues collected and amount of revenues invested in lower income populations are not affected by the pass-through rate, and a lower rate translates into less aggregate taxes paid by lower income populations, our net transfers to low-income communities is a lower-bound estimate. We focus the net transfer analysis on a dichotomous categorization of income since most programs targeted towards lower-income populations use a single cut point for defining eligibility. However, families with incomes above 200% FPL may still struggle to make ends meet and learning more about the tax impact in middle-income groups would be useful. We can only estimate the total amount of revenues targeted towards programs serving lower income populations and we are unable to estimate the direct financial benefit of these programs to individuals in this population. This is an important limitation because some people may receive direct or indirect benefits from targeted programs, while others may not. In addition, a dollar funding a targeted program may be worth more or less than a dollar in an individual’s pocket, depending on whether the multipliers of the investments are greater than or less than 1. Finally, our assessment is limited to examining volume of taxed beverages actually purchased post-tax implementation and does not quantify the degree to which these post-tax purchases may reflect changes from behavior pre-tax due to the increased price imposed by the tax. These changes could be differential by income, and thus it is possible that these taxes limit the choice set for lower income households to a greater degree than for higher income households. Similarly, if lower income households decide to travel further to purchase taxed beverages in a non-taxing jurisdiction, additional travel and time costs would be incurred and are unaccounted for in our analysis.

## 6. Conclusion

In conclusion, we examined three measures of sweetened beverage tax economic equity impacts. We found that the lowest-income households paid a larger proportion of household income on the tax, unsurprisingly. However, the absolute level of annual per capita dollar amount paid on taxes did not differ by household income. Furthermore, the dollar amount of revenue allocations targeted towards people with lower incomes exceeded the amount of tax collected from this income group, resulting in a net transfer of revenues paid by higher-income populations to programs targeted towards lower-income populations, in aggregate. Thus, when considering both population-level taxes paid and sufficiently targeted allocations of tax revenues, a sweetened beverage tax may be an equitable public policy.

## Supplementary Material

Supplemental Materials

## Figures and Tables

**Table 1:** Sample characteristics for Nielsen and OmniPanel participants in three cities with beverage taxes.

	Philadelphia	Seattle	San Francisco
	(N = 585)	(N = 212)	(N = 344)
	Unweighted N(weighted %) or Mean(SE)		
**Income**	$52,621 (2,058)	$78,642 (4,014)	$83,895 (3,222)
*Income category*			
Lowest Income, < 200% FPL	182 (36%)	55 (19%)	84 (17%)
Middle Income, 200–400% FPL	202 (31%)	63 (20%)	80 (16%)
Highest Income, >400% FPL	202 (32%)	95 (61%)	180 (66%)
*Race/ethnicity*			
Non-Hispanic White	318 (35%)	123 (63%)	72 (40%)
Non-Hispanic Black	155 (41%)	8 (6.8%)	12 (5.5%)
Non-Hispanic Asian	48 (9%)	57 (18%)	188 (33%)
Hispanic	50 (11%)	12 (7.7%)	39 (15%)
Non-Hispanic Other	15 (3.3%)	13 (4.6%)	33 (6.4%)
*Age category*			
Aged 21–24	14 (2.4%)	5 (2.6%)	12 (2.6%)
Aged 25–34	115 (21%)	63 (30%)	94 (22%)
Aged 35–44	164 (21%)	53 (20%)	97 (22%)
Aged 45–54	106 (15%)	40 (15%)	69 (15%)
Aged 55–64	110 (24%)	28 (19%)	43 (23%)
Aged 65+	76 (16%)	23 (13%)	29 (15%)
*Education category*			
Less than high school	23 (9.3%)	5 (2.7%)	12 (6.7%)
High school/GED	111 (37%)	24 (25%)	27 (12%)
Some college or completed technical school	182 (24%)	55 (23%)	97 (19%)
Completed college	180 (20%)	86 (31%)	150 (42%)
Completed graduate school	90 (9.3%)	43 (18%)	58 (20%)
*Household composition*			
Children under 18 Present	232 (28%)	47 (20%)	119 (19%)
Number of Household Members	2.5 (0.097)	2.1 (0.13)	2.2 (0.086)
**Per Capita Volume of Taxed Beverages (oz/yr.) by Income**	1,957 (150)	772 (128)	799 (113)
Lowest Income, < 200% FPL	2,069 (299)	1,085 (410)	546 (125)
Middle Income, 200–400% FPL	1,986 (225)	751 (220)	650 (137)
Highest Income, >400% FPL	1,803 (237)	681 (147)	902 (161)
**Per Capita Beverage Spending ($/yr.) by Income**	93 (6.9)	57 (8.3)	49 (5.2)
Lowest Income, < 200% FPL	98 (14)	77 (25)	38 (7.8)
Middle Income, 200–400% FPL	94 (10)	52 (12)	44 (7.9)
Highest Income, >400% FPL	85 (11)	53 (10)	53 (7.2)

Source/Notes: Data are authors’ calculations from Nielsen Homescan Panel and Numerator OmniPanel. Notes: Data are weighted to be representative of each city listed using raking weights, except for Ns, which are unweighted to show sample size.

**Table 2:** Per capita annual tax spending: Estimates from ordinary least squares regression of spending and log-transformed spending and predicted means.

	Philadelphia		Seattle		San Francisco	
Outcome	Log−transformed Tax paid per capita (exponentiated)	Tax paid per capita	Log−transformed Tax paid per capita (exponentiated)	Tax paid per capita	Log−transformed Tax paid per capita (exponentiated)	Tax paid per capita
*Panel A: Beta (95% Confidence Interval)*						
Lowest income (constant), <200% FPL	-Ref-	-Ref-	-Ref-	-Ref-	-Ref-	-Ref-
Middle income, 200−400% FPL	1.16 (0.78, 1.7)	−1.2 (−12, 9.8)	0.86 (0.40, 1.9)	− 5.8 (−22, 10)	1.42 (0.73, 2.7)	1.0 (−2.6, 4.7)
Highest income, >400% FPL	1.02 (0.68, 1.5)	− 4.0 (−15, 7.3)	0.61 (0.27, 1.32)	− 7.1 (−22, 7.9)	1.84 (0.98, 3.3)	3.6 (0.45, 7.6)
p−value for test of high income = middle income	0.47	0.58	0.28	0.79	0.28	0.24
*Panel B: Predicted (US dollars per year) based on regression estimates from Panel A*						
Lowest income	11 (8.2, 15)	31 (22, 40)	5.5 (3.0, 10)	19 (4.9, 33)	1.9 (1.1, 3.3)	5.5 (3.0, 7.9)
Middle income	13 (8.6, 19)	30 (24, 35)	4.7 (3.7, 8)	13.2 (−2.8, 29)	2.7 (1.4, 5.2)	6.5 (3.3, 9.8)
Highest income	11.2 (7.6, 17)	27 (16, 38)	3.4 (1.7, 18)	11.9 (−3.1, 27)	3.5 (1.9, 6.5)	9.1 (5.1, 13)
Observations	585	585	212	212	344	344

Source/Notes: Authors’ calculations based on Homescan and OmniPanel data. Top panel displays exponentiated regression coefficients for the log of dollars and dollars spent on the tax with income categories modeled as indicator variables. The lower-income group is the referent category; the coefficients for middle- and higheer-income groups are the difference in spending between each respective income group and the lower-income group. Confidence intervals on these coefficients that exclude the null value (0) indicate significant differences for middle or higher groups compared to lower income group. The p-value for the test that middle income and higheer income groups are different is shown in a separate row. For the outcomes that are log-transformed, exponentiating the coefficients for middle and higher income groups gives the relative difference between lower-income for that group. For instance, in Philadelphia, the beta/coefficient was 0.15; exponentiation of 0.15 equals 1.16, indicating that mean spending in the middle income group is 16% higher than that for the lower income group.

The bottom panel displays the estimates back transformed to predicted mean dollars for each income group. These are obtained through linear combinations of the relevant coefficients, and exponentiating these in the case of the log-transformed models.

**Table 3:** Per capita annual beverage and tax spending as proportion of household income: Estimates from ordinary least squared regressions of log-transformed spending on income categories.

	Philadelphia		Seattle		San Francisco	
	Log of proportion of HH income paid in taxes per capita (exponentiated)	Proportion of HH income paid in taxes per capita	Log of Proportion of HH income paid in taxes per capita (exponentiated)	Proportion of HH income paid in taxes per capita	Log of Proportion of HH income paid in taxes per capita (exponentiated)	Proportion of HH income paid in taxes per capita
*Panel A: Beta (95% Confidence Interval)*						
Lowest income (constant), < 200 %FPL	-Ref-	-Ref-	-Ref-	-Ref-	-Ref-	-Ref-
Middle income, 200−400 %FPL	0.33 (0.22, 0.52)	− 0.37 (−0.55, − 0.18)	0.27 (0.13, 0.51)	− 0.15 (−0.3, 0.00073)	0.46 (0.22, 0.91)	− 0.035 (−0.061, −0.0091)
Highest income, > 400 %FPL	0.15 (0.10, 0.22)	− 0.44 (−0.62, − 0.26)	0.08 (0.041, 0.17)	− 0.17 (−0.32, − 0.023)	0.25 (0.14, 0.46)	− 0.049 (−0.073, −0.024)
p−value for test of highest income=medium income	<0.01	<0.01	<0.01	<0.01	0.011	0.02
*Panel B: Predicted percent of income paid in beverage tax (US dollars per year)* b*ased on regression estimates from Panel A*						
Lowest income	0.17% (0.12, 0.22)	0.50% (0.32, 0.68)	0.07% (0.041, 0.12)	0.20% (0.045, 0.35)	0.02% (0.01, 0.04)	0.06% (0.039, 0.086)
Middle income	0.06% (0.036, 0.083)	0.13% (−0.055, 0.32)	0.02% (0.010, 0.040)	0.05% (−0.10, 0.20)	0.01% (0.0056, 0.022)	0.03% (0.0020, 0.054)
Highest income	0.02% (0.017, 0.036)	0.06% (−0.12, 0.24)	0.01% (0.003, 0.012)	0.03% (−0.12, 0.18)	0.01% (0.0033, 0.011)	0.01% (−0.010, 0.038)
Observations	585	585	212	212	344	344

Source/Notes: Author’s calculations based on Homescan and OmniPanel data. Panel A displays exponentiated regression coefficients for the log of dollars spent or dollars spent on the tax as a proportion of household income, with income categories modeled as indicator variables. The low-income group is the referent category, which is the constant term in these models. The coefficients for middle- and high-income groups are the difference in spending as a proportion of income between each income group and the low-income group. Confidence intervals that include the null value (0) indicate that the estimates are not statistically significantly different from estimate for the low-income group. Since the outcomes are log-transformed, exponentiating the coefficients for middle and high income gives the relative difference between low-income and that income group. For instance, in Philadelphia, the coefficient for the middle income group was –1.1; exponentiation of this coefficient equals 0.33, indicating that mean spending in the middle income group is 33% of the value for the lowest income group (or stated another way, 67% lower than the lowest income group (1–0.33 = 0.67)).

Panel B displays the estimates back transformed to predicted proportion of income for each income group. These are obtained through exponentiating the linear combination of relevant coefficients.

**Table 4:** Estimated aggregate amount of tax paid by lower and higher income populations compared to amount of tax revenue targeted towards each population in each city.

	Tax Paid					Tax Revenue Allocations	
	A	B	C	D	E	G	H
	Average tax paid per capita	Population (N)	Estimated aggregate total paid on taxes for purchases included in panel data (A*B)^1^	Proportion of estimated aggregate total paid^2^ (C_incomei_/C_total_)	Total revenue collected (total) and scaled-up estimated tax paid by income^3^ (by income: D*total revenue)	Dollars allocated to programs, total and by income (%of total revenues)^4,5^	Net transfer (aggregate amount paid minus amount allocated)^6^ (G-E)
**Philadelphia**		1,532,157	$45,351,452	1.00	$75,122,000	$72,796,314 (97%)	
Lowest income	$31.0	700,196	$21,726,799	0.48	$35,989,158	$52,413,346 (70%)	$16,424,188
Higher income	$28.4	831,961	$23,624,653	0.52	$39,132,842	$20,382,968 (27%)	
**Seattle**		744,949	$10,095,739	1.00	$22,254,000	$15,627,717 (70%)	
Lowest income	$19.0	146,755	$2,785,854	0.28	$6,140,847	$12,502,174 (56%)	$6,361,327
Higher income	$12.2	598,194	$7,309,885	0.72	$16,113,153	$3,125,543 (14%)	
**San Francisco**		883,305	$7,016,276	1.00	$13,181,608	$10,530,336 (80%)	
Lowest income	$5.5	189,911	$1,036,652	0.15	$1,947,578	$7,265,932 (55%)	$5,318,354
Higher income	$8.5	702,227	$5,979,624	0.85	$11,234,030	$3,264,404 (25%)	

Source/Notes: Authors’ calculations from American Community Survey data, Nielsen’s Homescan, Numerator’s OmniPanel, administrative documents of revenues collected and program allocations from all three cities.

1 We used the weighted mean per capita spending on the tax in a dichotomous version of income groups (<200% FPL vs ≥200% FPL) and multiplied this by the number of people in each city (from the American Community Survey) in each income group to approximate total aggregate amount paid in beverage tax for each income group.

2 We calculated the proportion of the total taxes paid on purchases captured by the panel data according to income.

3 We then use this proportion to scale up to the predicted amount of tax contributed by each income group to the actual total tax revenue collected.

4 We did not include revenues allocated for administration, evaluation, or unspent funds in the calculation of revenues targeted towards lower income populations. The difference between the total allocated and total revenues is administrative, evaluation, or unspent: Philadelphia: $ 2,325,686; Seattle: $6,626,283; San Francisco: $3,141,608.

5 Philadelphia and San Francisco allocated more funds than total revenue in the first year.

6 To calculate the net transfer of funds between lower- and higher-income populations, we subtracted the aggregate predicted amount spent on the tax by the lower-income population from the amount of tax revenues spent on programs targeting lower-income populations. If this number is positive, it indicates an aggregate transfer from higher-income groups to lower-income groups and vice versa if it is negative.

## References

[R1] Allcott H, Lockwood BB, Taubinsky D, 2019. Regressive Sin Taxes, with an Application to the Optimal Soda Tax . Q J. Econ 134 (3), 1557–1626. 10.1093/qje/qjz017.

[R2] Backholer K, Sarink D, Beauchamp A, Keating C, Loh V, Ball K, Martin J, Peeters A, 2016. The impact of a tax on sugar-sweetened beverages according to socio-economic position: a systematic review of the evidence. Public Health Nutr. 19 (17), 3070–3084. 27182835 10.1017/S136898001600104X PMC10270974

[R3] Bollinger BK, Sexton S, 2018. Local Excise Taxes, Sticky Prices, and Spillovers: Evidence from Berkeley’s Soda Tax. SSRN Electron J. 10.2139/ssrn.3087966.

[R4] Cawley J, Frisvold D, Hill A, Jones D, 2019. The impact of the Philadelphia beverage tax on purchases and consumption by adults and children. J. Health Econ 67, 102225. 10.1016/j.jhealeco.2019.102225. 31476602

[R5] Cawley J, Frisvold D, Hill A, Jones D, 2020. Oakland’s sugar-sweetened beverage tax: Impacts on prices, purchases and consumption by adults and children. Econ. Hum. Biol 37, 100865. 10.1016/j.ehb.2020.100865. 32126505

[R6] Chi DL, Scott JAM, 2019. Added sugar and dental caries in children: A scientific update and future steps. Dent. Clin. North Am 63 (1), 17–33. 10.1016/j.cden.2018.08.003. 30447790 PMC6242348

[R7] Falbe J, 2020. The ethics of excise taxes on sugar-sweetened beverages. Physiol. Behav 225, 113105. 10.1016/j.physbeh.2020.113105. 32712210 PMC7377978

[R8] Falbe J, Lee MM, Kaplan S, Rojas NA, Ortega Hinojosa AM, Madsen KA, 2020. Higher sugar-sweetened beverage retail prices after excise taxes in Oakland and San Francisco. Am. J. Public Health 110 (7), 1017–1023. 10.2105/AJPH.2020.305602. 32437271 PMC7287565

[R9] Jones-Smith JC, Pinero Walkinshaw L, Oddo VM, Knox M, Neuhouser ML, Hurvitz PM, Saelens BE, Chan N, 2020. Impact of a sweetened beverage tax on beverage prices in Seattle, WA. Econ. Hum. Biol 39, 100917. 10.1016/j.ehb.2020.100917. 32801099 PMC11316513

[R10] Kane RM, Malik VS, 2019. Understanding beverage taxation: Perspective on the philadelphia beverage tax’s novel approach. J. Public Health Res 8 (1466), 40–45. 10.4081/jphr.2019.1466. PMC6478003 31044136

[R11] Lee MM, Falbe J, Schillinger D, Basu S, McCulloch CE, Madsen KA, 2019. Sugar-Sweetened Beverage Consumption 3 Years After the Berkeley, California, Sugar-Sweetened Beverage Tax. Am. J. Public Health 109 (4), 637–639. 10.2105/AJPH.2019.304971. 30789776 PMC6417561

[R12] Luger M, Lafontan M, Bes-Rastrollo M, Winzer E, Yumuk V, Farpour-Lambert N, 2017. Sugar-sweetened beverages and weight gain in children and adults: A systematic review from 2013 to 2015 and a comparison with previous studies. Obes Facts 10 (6), 674–693. 10.1159/000484566. 29237159 PMC5836186

[R13] Malik VS, Popkin BM, Bray GA, Després JP, Hu FB, 2010. Sugar-sweetened beverages, obesity, type 2 diabetes mellitus, and cardiovascular disease risk. Circulation. 121 (11), 1356–1364. 10.1161/CIRCULATIONAHA.109.876185. 20308626 PMC2862465

[R14] Malik VS, Li Y, Pan A.n., De Koning L, Schernhammer E, Willett WC, Hu FB, 2019. Long-term consumption of sugar-sweetened and artificially sweetened beverages and risk of mortality in US adults. Circulation 139 (18), 2113–2125. 10.1161/CIRCULATIONAHA.118.037401. 30882235 PMC6488380

[R15] Manning WG, Mullahy J, 2001. Estimating log models: to transform or not to transform? J. Health Econ 20 (4), 461–494. 10.1016/S0167-6296(01)00086-8. 11469231

[R16] McDowell DR, Allen-Smith JE, McLean-Meyinsse PE, 1997. Food expenditures and socioeconomic characteristics: focus on income class. Am. J. Agric. Econ 79 (5), 1444–1451. 10.2307/1244359.

[R17] Popkin BM, Ng SW, 2021. Sugar-sweetened beverage taxes: Lessons to date and the future of taxation. PLoS Med. 18 (1), 1–6. 10.1371/journal.pmed.1003412. PMC7790261 33411708

[R18] Powell LM, Marinello S, Leider JAT, 2021. A Review and Meta-Analysis of the Impact of Local U.S. Sugar-Sweetened Beverage Taxes on Demand Chicago, IL. https://p3rc.uic.edu/.

[R19] Powell LM, Leider J, 2020. The impact of Seattle’s Sweetened Beverage Tax on beverage prices and volume sold. Econ. Hum. Biol 37, 100856. 10.1016/j.ehb.2020.100856. 32070906

[R20] Roberto CA, Lawman HG, LeVasseur MT, Mitra N, Peterhans A, Herring B, Bleich SN, 2019. Association of a Beverage Tax on Sugar-Sweetened and Artificially Sweetened Beverages with Changes in Beverage Prices and Sales at Chain Retailers in a Large Urban Setting. JAMA – J. Am. Med. Assoc 321 (18), 1799. 10.1001/jama.2019.4249. PMC6518342 31087022

[R21] Saksena MJ, Okrent AM, Anekwe TD, , 2018. America’s Eating Habits: Food Away From Home. https://www.ers.usda.gov/publications/pub-details/?pubid=90227. Accessed May 3, 2021.

[R22] Silver LD, Ng SW, Ryan-Ibarra S, Taillie LS, Induni M, Miles DR, Poti JM, Popkin BM, Langenberg C, 2017. Changes in prices, sales, consumer spending, and beverage consumption one year after a tax on sugar-sweetened beverages in Berkeley, California, US: A before-and-after study. PLoS Med. 14 (4), e1002283. 10.1371/journal.pmed.1002283. 28419108 PMC5395172

[R23] Smith TA, Lin BW, Lee J-Y, 2010. Taxing caloric sweetened beverage: Potential effects on beverage consumption, calorie intake, and obesity. Econ. Res. Rep July (100): 25. doi:10.2139/ssrn.2118636.

[R24] U.S. Department of Health and Human Services. Prior HHS Poverty Guidelines and Federal Register References. https://aspe.hhs.gov/prior-hhs-poverty-guidelines-and-federal-register-references. Accessed April 27, 2021.

[R25] Urban Institute Brookings Institution. Brief Description of the Tax Model. Tax Policy Center Resources. https://www.taxpolicycenter.org/resources/brief-description-tax-model. Published 20Accessed April 20, 2021.

[R26] Zhen C, Finkelstein EA, Nonnemaker JM, Karns SA, Todd JE, 2014. Predicting the effects of sugar-sweetened beverage taxes on food and beverage demand in a large demand system. Am. J. Agric. Econ 96 (1), 1–25. 10.1093/ajae/aat049. 24839299 PMC4022288

[R27] Zhong Y, Auchincloss AH, Lee BK, Kanter GP, 2018. The Short-Term Impacts of the Philadelphia Beverage Tax on Beverage Consumption. Am. J. Prev. Med 55 (1), 26–34. 10.1016/j.amepre.2018.02.017. 29656917

