# Supplementary material for: Sweetened beverage taxes: Economic benefits and costs according to household income
Source: Food Policy. Author manuscript; Available in PMC 2023 Nov 29. (PMC10686549; doi:10.1016/j.foodpol.2022.102277)
Supplement: Supplemental Materials [file NIHMS1895703-supplement-Supplemental_Materials.docx]

Supplemental Materials

Details of Data Harmonization, Missingness and Imputation

*Income*. The categories of household income are different in the two datasets; we keep the original categories for each when assigning midpoints, then picked aggregate values that we could make consistent across the two data sets: <200% FPL, 200-400% FPL, >400% FPL.

For all variables used in creating weights to make the sample representative, categories were harmonized across data sets.

The Nielsen data included the following income ranges: 0-5,000, 5,000-7,999, 8,000-9,999, 10,000-11,999, 12,000-14,999, 15000-19,999, 20,000-24,999, 25,000-29,999, 30,000-34,999, 35,000-39,999, 40,000-44,999, 45,000-49,999, 50,000-59,999, 60,000-69,999, 70,000-99,999, 100-124,999, 125,000-149,000, 150-199, >200K.

The Omni panel data included the following income ranges: 0-19,999, 20,000-39,999, 40,000-59,999, 60,000-79,999, 80,000-99,999, 100,000-124,999, >125,000

*Missingness.* The OmniPanel data was missing information on beverage volume for 48% (n = 15016) of the observations.

*Imputation.* We used hot deck imputation to impute the missing beverage volume values.(1–3) The hot deck imputation selects observations that match the missing observation on sets of variables creating decks of similar observations. Each collection of similar observation is the hot deck. A value for the missing observation is then randomly selected from the deck of similar observations. This approach is akin to nearest neighbor imputation but works well with categorical data.(3) In addition, the missing data in this case stems largely from differing ways that stores print receipts (i.e. some including the volume of each beverage and/or GTIN and others not so we can reasonably assume that the data are missing completely at random (MCAR). In this analysis we successfully imputed values for 63% of missing observations using the hot deck imputation approach. Briefly, we iterated over a series of sequentially less restrictive deck selection criteria each of which generated a hot deck of similar observations. The first set of iterations selected a deck from observations from the same household if that household had at least three purchases of a beverage of the same beverage type (e.g., soda), volume unit (e.g., fl. oz., liter), brand (e.g., Pepsi, Lipton Brisk, Rockstar), store type (e.g. supermarket) and store (e.g., Ralph’s) as the beverage purchase with missing volume. Progressively the requirement for matching on store and store type were relaxed so that items only needed to match on beverage type, brand, and volume unit within the household, if a match on the other characteristics had not yet been located. We were able to impute 2266 (16%) of the missing values with this highly restrictive inclusion criteria. We then further relaxed the similarity required for inclusion in the deck matching on the purchase and household characteristics, but we required five or more observations to make a deck for sampling. The beverage purchase characteristics included beverage type, brand, volume unit, store type and store while the household characteristics included age/generation, income, education, gender, and race/ethnicity. We sequentially iterated over these characteristics first matching on all four purchase characteristics and on all five household characteristics, then four, and then three household characteristics while preserving all characteristics about the purchase itself. The age/generation household variable was never excluded across this set of iterations, which increased the fraction of imputed values for missing observations to 18%. This iterative process was repeated with the variable for beverage brand excluded, which increased the fraction of imputed values to 37%. Next the iteration was repeated dropping of the required match on the store but adding the brand match back to the inclusion criteria, imputing 56% of the missing data. Finally, the product brand and the store were excluded leaving the type of beverage and the volume units as the beverage characteristics. The demographic variables were then cycled through, beginning with the same household followed by matching on at least 5, then 4, and finally 3 of the following (income, education, age, gender, race and ethnicity). Income was always included as a matching criterion. This final imputation increased the fractions of imputed values to 63% of the missing responses. In a test of this procedure we were able to recapture 98% of a randomly deleted 15% of our observed volume observations, with 47% of the imputed values being identical to the known but deleted volume values. The imputed volumes also had a median error of zero ounces when compared to the true values.

Supplemental Figure 1 display the distributions of actual versus imputed volume for each of the three income categories. To appropriately account for the uncertainty in the imputation, we imputed 5 different realizations of the missing data. We then perform all of our statistical summaries and analyses on each of the five different datasets.

*Estimation with Imputation.* To produce estimates that combine the Nielsen and OmniPanel data sets, we combine the Nielsen dataset with each of the OmniPanel datasets, apply the population weights, and perform the analyses on the combined Nielsen and OmniPanel data (with 5 different imputation realizations each combined with the same variables from Nielsen) and then we take the average of the estimates from each of the five results.

References

1. Roth PL. Missing Data: A Conceptual Review for Applied Psychologists. Pers Psychol. 1994 Sep 1;47(3):537–60.

2. Schafer JL. Multiple imputation: a primer. Stat Methods Med Res. 1999 Feb 2;8(1):3–15.

3. Andridge RR, Little RJA. A review of hot deck imputation for survey non-response. Int Stat Rev. 2010 Apr;78(1):40–64.

| **Supplemental Table 1. Unweighted sample characteristics for Nielsen and Omni panelists in three cities with beverage taxes** | | | | | | | | | |  |
| --- | --- | --- | --- | --- | --- | --- | --- | --- | --- | --- |
|  | **Philadelphia** | | | **Seattle** | | | **San Francisco** | | | |
|  | **Nielsen** | **Omni** | **Nielsen + Omni** | **Nielsen** | **Omni** | **Nielsen + Omni** | **Nielsen** | **Omni** | **Nielsen + Omni** | |
|  | **N (%) or Mean (SE)** | | | | | | | | | |
| Estimated Income | 55051 (2435) | 60706 (1906) | 59017 (1524) | 65516 (4176) | 63500 (3643) | 64096 (2842) | 78686 (4122) | 73252 (2722) | 74484 (2303) | |
| Low-Income | 50 (32%) | 132 (32%) | 182 (31%) | 9 (9.2%) | 46 (31%) | 55 (26%) | 11 (15%) | 73 (27%) | 84 (24%) | |
| Middle-Income | 68 (42%) | 134 (33%) | 202 (34%) | 22 (39%) | 41 (27%) | 63 (30%) | 19 (33%) | 61 (23%) | 80 (23%) | |
| High-Income | 57 (26%) | 145 (35%) | 202 (34%) | 32 (52%) | 63 (42%) | 95 (45%) | 48 (52%) | 132 (50%) | 180 (52%) | |
| Non-Hispanic White | 86 (36%) | 232 (56%) | 318 (54%) | 46 (64%) | 77 (51%) | 123 (58%) | 27 (37%) | 45 (17%) | 72 (21%) | |
| Non-Hispanic Black | 66 (40%) | 89 (22%) | 155 (26%) | 2 (5.3%) | 6 (4%) | 8 (3.8%) | 4 (4.1%) | 8 (3%) | 12 (3.5%) | |
| Non-Hispanic Asian | 5 (5.2%) | 43 (10%) | 48 (8.2%) | 11 (19%) | 46 (31%) | 57 (27%) | 25 (30%) | 163 (61%) | 188 (55%) | |
| Hispanic | 14 (13%) | 36 (8.8%) | 50 (8.5%) | 3 (9.5%) | 9 (6%) | 12 (5.6%) | 11 (18%) | 28 (11%) | 39 (11%) | |
| Non-Hispanic Other | 4 (5.2%) | 11 (2.7%) | 15 (2.6%) | 1 (2.1%) | 12 (8%) | 13 (6.1%) | 11 (11%) | 22 (8.3%) | 33 (9.6%) | |
| Aged 21-24 | 1 (1.5%) | 13 (3.2%) | 14 (2.4%) | 1 (1.8%) | 4 (2.7%) | 5 (2.4%) | 0 (0%) | 12 (4.5%) | 12 (3.5%) | |
| Aged 25-34 | 14 (22%) | 101 (25%) | 115 (20%) | 10 (31%) | 53 (36%) | 63 (30%) | 6 (25%) | 88 (33%) | 94 (27%) | |
| Aged 35-44 | 28 (17%) | 136 (33%) | 164 (28%) | 10 (14%) | 43 (29%) | 53 (25%) | 11 (8.2%) | 86 (32%) | 97 (28%) | |
| Aged 45-54 | 27 (19%) | 79 (19%) | 106 (18%) | 12 (21%) | 28 (19%) | 40 (19%) | 21 (29%) | 48 (18%) | 69 (20%) | |
| Aged 55-64 | 56 (22%) | 54 (13%) | 110 (19%) | 13 (12%) | 15 (10%) | 28 (13%) | 18 (19%) | 25 (9.4%) | 43 (13%) | |
| Aged 65+ | 49 (19%) | 27 (6.6%) | 76 (13%) | 17 (20%) | 6 (4%) | 23 (11%) | 22 (19%) | 7 (2.6%) | 29 (8.4%) | |
| Less than high school | 6 (7.9%) | 17 (4.1%) | 23 (3.9%) | 0 (0%) | 5 (3.3%) | 5 (2.3%) | 1 (5.5%) | 11 (4.1%) | 12 (3.5%) | |
| High school/GED | 46 (36%) | 65 (16%) | 111 (19%) | 12 (27%) | 12 (8%) | 24 (11%) | 3 (13%) | 24 (9%) | 27 (7.8%) | |
| Some college or completed technical school | 46 (26%) | 136 (33%) | 182 (31%) | 12 (23%) | 43 (29%) | 55 (26%) | 19 (18%) | 78 (29%) | 97 (28%) | |
| Completed college | 58 (24%) | 122 (30%) | 180 (31%) | 24 (29%) | 62 (41%) | 86 (40%) | 37 (44%) | 113 (42%) | 150 (44%) | |
| Completed graduate school | 19 (6.4%) | 71 (17%) | 90 (15%) | 15 (22%) | 28 (19%) | 43 (20%) | 18 (20%) | 40 (15%) | 58 (17%) | |
| Children under 18 Present | 36 (28%) | 196 (48%) | 232 (40%) | 6 (24%) | 41 (27%) | 47 (22%) | 9 (23%) | 110 (41%) | 119 (35%) | |
| Number of Household Members | 2.2 (0.11) | 3 (0.081) | 2.7 (0.067) | 1.7 (0.12) | 2.5 (0.13) | 2.2 (0.1) | 2.1 (0.15) | 3 (0.097) | 2.8 (0.085) | |

| **Supplemental Table 2. Estimates from regression coefficients and predicted means for amounts spent on taxed beverages and on the tax per capita, separating Nielsen and OmniPanel** | | | | | | | |
| --- | --- | --- | --- | --- | --- | --- | --- |
|  | **Philadelphia** | | **Seattle** | | | **San Francisco** | |
|  | **Tax paid per capita** | | **Tax paid per capita** | | | **Tax paid per capita** | |
|  | **Nielsen** | **Omni** | | **Nielsen** | **Omni** | **Nielsen** | **Omni** |
|  |  | | | | | | |
| Higher-income | -0.29  (-1, 0.44) | 0.45  (-0.12, 1) | | -2  (-4.1, 0.077) | 0.33  (-0.55, 1.2) | 0.022  (-1.4, 1.4) | 0.66  (-0.13, 1.5) |
| Middle-income | 0.18  (-0.55, 0.92) | 0.34  (-0.25, 0.93) | | -1.3  (-3.2, 0.61) | 0.45  (-0.38, 1.3) | 0.31  (-0.88, 1.5) | 0.69  (-0.31, 1.7) |
| Low-income (constant) | Ref | Ref | | Ref | Ref | Ref | Ref |
|  |  | | | | | | |
| Low-income total | 8.17 | 11.02 | | 14.88 | 2.72 | 1.73 | 1.84 |
| Middle income total | 9.78 | 15.49 | | 4.06 | 4.26 | 2.36 | 3.67 |
| High income total | 6.11 | 17.29 | | 2.01 | 3.78 | 1.77 | 3.56 |
| Low Income/High Income | 1.34 | 0.64 | | 7.39 | 0.72 | 0.98 | 0.52 |
| Observations | 175 | 410 | | 63 | 149 | 78 | 266 |
| p-value for test of high income= medium income | 0.19 | 0.69 | | 0.29 | 0.78 | 0.65 | 0.92 |

| **Supplemental Table 3. Estimates from regression coefficients and predicted means for amounts spent on taxed beverages and on the tax by household income, separating Nielsen and OmniPanel** | | | | | | |
| --- | --- | --- | --- | --- | --- | --- |
|  | **Philadelphia** | | **Seattle** | | **San Francisco** | |
|  | **Tax paid per capita** | | **Tax paid per capita** | | **Tax paid per capita** | |
|  | **Nielsen** | **Omni** | **Nielsen** | **Omni** | **Nielsen** | **Omni** |
|  |  | | | | | |
| Higher-income | -2.1  (-2.8, -1.3) | -1.6  (-2.1, -1) | -3.4  (-5.7, -1.1) | -1.9  (-2.7, -1.1) | -1.5  (-2.7, -0.26) | -1.3  (-2.1, -0.46) |
| Middle-income | -0.82  (-1.6, -0.085) | -1  (-1.6, -0.41) | -1.9  (-3.9, 0.14) | -0.79  (-1.5, -0.075) | -0.034  (-1.4, 0.75) | -0.47  (-1.5, 0.59) |
| Low-income (constant) | Ref | Ref | Ref | Ref | Ref | Ref |
|  |  | | | | | |
| Low-income total | 0.10 | 0.18 | 0.10 | 0.05 | 0.01 | 0.02 |
| Middle-income total | 0.04 | 0.07 | 0.01 | 0.02 | 0.01 | 0.02 |
| High-income total | 0.01 | 0.04 | 0.00 | 0.01 | 0.00 | 0.01 |
| Low-Income/High-Income | 8.17 | 4.95 | 29.96 | 6.69 | 4.48 | 3.67 |
| Observations | 175 | 410 | 63 | 149 | 78 | 266 |
| p-value for test of high income=  medium income | 0.0013 | 0.047 | 0.041 | 0.01 | 0.05 | 0.037 |
